# Supplementary material for: Low-dose lipopolysaccharide inhibits spinal cord injury-induced neuronal apoptosis by regulating autophagy through the lncRNA MALAT1/Nrf2 axis
Source: PeerJ. 2023 Aug 28;11:e15919. doi: 10.7717/peerj.15919 (PMC10470450; doi:10.7717/peerj.15919)
Supplement: Supplemental Information 4 — Retouching certificate [file peerj-11-15919-s004.pdf]

# Bullet Edits

## EDITORIAL CERTIFICATE

This document certifies that the paper listed below was edited and proofread for proper English language, grammar, punctuation, spelling, and overall style by one or more than one highly qualified native speakers at Bullet Edits. All of the suggested amendments were tracked with the Microsoft Word "Track Changes" feature. Therefore, the author had the option to reject or accept each change individually.

## Manuscript Title:

Low-dose lipopolysaccharide inhibits spinal cord injury-induced neuronal apoptosis by regulating autophagy through the lncRNA MALAT1/Nrf2 axis

## Anti-counterfeiting Code:

b51d9146baa90ac5b288bd73de5b08f1

## Date Issued:

2022-12-20 21:58:51

Bullet Edits is a registered company headquartered in the UK with a global presence. We offer a range of editing, proofreading services to authors. Our Ph.D. editors are all native English speakers from the USA and UK. Authors who work with Bullet Edits are guaranteed excellent language quality and timely delivery.

Address: BULLET EDITS LIMITED, 85 Great Portland Street, London,UK
Tel: UK. (+44)20457 70286 / US.(+1)312-313-9179
Web: www.bulletedits.cn/Email: info@bulletedits.cn
VAT: GB 378 9316 43

BULLET EDITS LIMITED ★ COMPANY NO. 13345261 SEAL ★ ENGLAND AND WALES
